# Relationship between video game addiction and bladder/bowel dysfunction in children

**DOI:** 10.7705/biomedica.7018

**Published:** 2024-08-29

**Authors:** Öznur Tiryaki, Dilek Menekşe, Nursan Çinar

**Affiliations:** 1 Department of Midwifery, Faculty of Health Sciences, Sakarya University, Sakarya, Turkey Sakarya University Sakarya University Sakarya Sakarya; 2 Department of Pediatric Nursing, Faculty of Health Sciences, Sakarya University, Sakarya, Turkey Sakarya University Sakarya University Sakarya Sakarya

**Keywords:** Video games, technology addiction, urinary bladder diseases, child, juegos de video, adicción a la tecnología, enfermedades de la vejiga urinaria, niño

## Abstract

**Introduction.:**

Video games have a strong influence on children and adolescents. Video game addiction has negative effects on children’s health.

**Objective.:**

To determine the relationship between video game addiction and bladder/bowel dysfunction in children.

**Materials and methods.:**

Three hundred sixty-three children and their mothers who met the inclusion criteria constituted the sample of this correlational study. The data were collected using a descriptive information form, the Video Game Addiction Scale for Children, and the Childhood Bladder and Bowel Dysfunction Questionnaire.

**Results.:**

We found that 72.5% of the children were nine years old and 27.5% were ten years old; 50.4% were males and 49.6% were female. While 4.7% of the children who participated in the study were underweight, 19.6% were overweight, and 17.9% were obese. The mean Video Game Addiction Scale for Children score was 50.77 ± 16.17, whereas the mean Childhood Bladder and Bowel Dysfunction Questionnaire score was 29.98 ± 8.90. The ratio of children with a mean Video Game Addiction Scale for Children scores equal to or greater than 90 was 0.8% (n = 3). We found that 3.6% (n = 13) of the children had urinary/fecal incontinence while playing video games. There was a weak positive relationship between the dimensions of the Video Game Addiction Scale for Children scores, the Childhood Bladder and Bowel Dysfunction Questionnaire scores, and children’s bladder and bowel function (r = 0.220; p < 0.05).

**Conclusions.:**

There is a correlation between children’s video game addiction level and their bladder and bowel dysfunction grade. Higher video game addiction levels correspond to higher bladder and bowel dysfunction.

## Resumen

**Introducción.:**

Los videojuegos tienen una fuerte influencia en los niños y adolescentes. La adicción a ellos tiene efectos perjudiciales para la salud de los niños.

**Objetivo.:**

Determinar la relación entre la adicción a los videojuegos y la disfunción vesical o intestinal en niños.

**Materiales y métodos.:**

Los 373 niños y sus madres que cumplieron con los criterios de inclusión constituyeron la muestra de este estudio descriptivo y correlacional. Los datos se recopilaron mediante un formulario de información descriptiva, la *Video Game Addiction Scale for Children* y el *Childhood Bladder and Bowel Dysfunction Questionnaire*.

**Resultados.:**

El 72,5 % de los niños tenía nueve años y, el 27,5 %, diez; el 50,4 % eran hombres y el 49,6 %, mujeres. El 4,7 % de los estudiantes que participaron en el estudio tenían bajo peso, el 19,6 % presentaba sobrepeso y el 17,9 % tenía obesidad. Se constató que las puntuaciones medias de las escalas fueron 50,77 ± 16,17 para la *Video Game Addiction Scale for Children* y 29,98 ± 8,90 para el *Childhood Bladder and Bowel Dysfunction Questionnaire*. La proporción de niños con una puntuación de 90 o superior en la dicha escala fue del 0,8 % (n = 3). Se encontró que el 3,6 % (n = 13) de los niños tenía incontinencia urinario-fecal mientras jugaba videojuegos. Se reveló que había una correlación positiva débil entre las dimensiones de las dos escalas evaluadas, y la función de la vejiga y el intestino (p < 0,05).

**Conclusiones.:**

El grado de adicción a los videojuegos de los niños está correlacionado con su grado de disfunción vesical o intestinal: entre mayor sea la adicción, mayor es la disfunción.

The video games industry, which attracts people of all ages, genders, and social classes, is widespread worldwide with products that run on mobile phones and computers. The intensive performance of gaming activities may turn into an addiction ^1^. Video games have a strong influence on children and adolescents. They affect gamers’ lifestyle, mentality, habits, and preferences to be alone ^2^. Children who spend an hour or more on a computer/video game per day are at a high risk of dysfunctional eating habits ^3^. This situation also affects voiding and bowel disorders.

The prevalence of voiding disorders has increased in recent years ^4^. The incidence of urinary incontinence in children is between 9.01 and 21.7% ^4^^-^^6^. The close relationship between voiding and bowel disorders is well known. For this reason, the expression “bowel/bladder dysfunction” is used to cover both groups of conditions: functional bladder (excessive activity, frequent urination) and bowel (constipation, fecal incontinence) problems ^7^.

The most common problem among bowel disorders is constipation, varying in children between 4.7 and 28.7% ^8^^-^^10^. Functional constipation depends on many factors, like genetics, socioeconomic status, maternal education levels, and nutritional habits ^11^. These problems adversely affect the child’s quality of life, school success, absenteeism, and learning ^12^^,^^13^. Based on this information, health professionals providing healthcare to children play a significant role in identifying children at risk to prevent diseases that may adversely affect their lives, aligning with the holistic care philosophy and providing education and care according to their needs.

The adverse effect of video game addiction on physical health has been clearly explained in the literature. However, no reported study has evaluated the relationship between these two conditions (video game addiction and bladder/bowel dysfunction).

This study aimed to assess the relationship between video game addiction and bladder/bowel dysfunctions in children. The research questions were: What are the factors affecting video game addiction? What are the factors affecting bladder and bowel dysfunction? Is there a relationship between video game addiction and bladder and, bowel dysfunction in children?

## Materials and methods

### Study design

This study was conducted with a correlational design.

### Sample and population

The study was conducted in four schools affiliated to the Serdivan District National Education Directorate located in the district center of Sakarya province in the Marmara region of Turkey, between 17/10/2020 and 17/11/2020.

A total of 1,179 students in the third (n = 566) and fourth grade (n = 613) of four primary schools, randomly selected among eight schools, constituted the population.

The sample size was calculated according to the formula for a known population. The number of participants was estimated as n = 290 using Neyman allocation, a stratified random sampling method. We applied the stratum sample size formula*n, and the number of students in third grade to be included was n = 139 (school A = 31, school B = 58, school C = 29, school D = 21), and the number of students in fourth grade to be included was n = 151 (school A = 37, school B = 64, school C = 28, school D = 22). The number of samples determined according to grade strata was proportionally allocated among the schools.

The sample consisted of 363 students and their mothers who met the inclusion criteria, voluntarily accepted to participate in the study, and filled out the data collection forms.

The inclusion criteria were: a) Students aged between nine and ten years, b) No involvement in an inclusive program, c) Students living with at least one parent, d) Mother’s and child’s willingness to participate in the study, e) No communication problem in the mother or the child, and f) Completion of the data collection forms by the mother and child.

### Data collection instruments

In the study, the data were collected using a descriptive information form, the Video Game Addiction Scale for Children (VASC), and the Childhood Bladder and Bowel Dysfunction Questionnaire (CBBDQ).

*Descriptive information form:* This form was prepared by the researchers to determine the characteristics of children and their families and consisted of ten questions. These questions were about descriptive characteristics such as the ages of the child and their parents, gender, video game playing behaviors, parental education and employment status, economic status, family type, and the number of living children in their family.

*Video Game Addiction Scale for Children (VASC):* This scale was developed and tested for validity and reliability by Yilmaz et al., and it is a five-point Likert-type rating scale ^14^. The scale consists of four dimensions (self-control, reward/reinforcement, problems, and involvement) and 21 items. VASC is applied to children between 9 and 12 years old. Total scores are obtained by summing the scores of all items. The total score to be obtained from the scale varies between 21 and 105. A score above 90 indicates a possible addiction to video games.

The Cronbach’s alpha internal consistency coefficient for the Turkish form of the scale was reported as 0.89. The reliability analyses of the scale in Turkey revealed sufficient levels of reliability for the overall scale and its dimensions. While the Cronbach’s alpha coefficient for the overall scale for this study sample was 0.93, the coefficients for the dimensions of the scale were 0.91 for self-control, 0.79 for reward/reinforcement, 0.80 for problems, and 0.66 for involvement.

*Childhood Bladder and Bowel Dysfunction Questionnaire (CBBDQ):* This questionnaire was developed by van Engelenburg-van Lonkhuyzen *et al*. (2017) and tested for validity and reliability by Aydin *et al*. (2020) in Turkey; it consists of 18 items and two dimensions: (1) bladder subscale (10 items) and (2) bowel subscale, including abdominal pain and bloated belly (8 items) ^15^^,^^16^. CBBDQ is used for children between 5 and 12 years old and is a five- point Likert-type scale in which each item ranges from 0 to 4. Higher CBBDQ scores are interpreted as the presence of more symptoms.

The Cronbach’s alpha value of the Turkish form of the scale was reported as 0.83, which showed good internal consistency ^16^. The Cronbach’s alpha of the scale in this study sample was 0.74.

### Statistical analysis

We contacted the administrators of the randomly selected schools and explained to them the aim of the study, the classes in which the data would be collected, and the time interval. The purpose of the study was detailed to the children and their parents. We sent the latter an explanatory text regarding the aim of the study, its confidentiality, and how the collected data would be used. The text included the phone number of the researcher to answer any questions the participants might have. Written informed consent was signed by the mothers who were previously aware of data safety. Mothers who did not want to participate in the study were excluded. The Descriptive Information Form and the CBBDQ were filled out by the mothers and their children, and the VASC was completed by the children only.

The collected data were analyzed using IBM SPSS™ Statistics, version 23, and IBM SPSS AMOS™, version 23. Frequency distributions were given for the categorical variables, and descriptive statistics (mean, standard deviation, minimum, and maximum) were presented for the numeric variables.

For the normally distributed data, an independent-sample t test was used to examine the differences between categorical variables in two groups, and a one-way analysis of variance (ANOVA) was used to compare more than two groups. For the non-normally distributed data, the Mann-Whitney U test was used to compare two groups, and the Kruskal-Wallis test was used to compare more than two groups.

Tamhane’s T2, Tukey’s ANOVA, and Games-Howell tests were used for post hoc analyses. Pearson’s correlation coefficients were calculated to determine correlations between the VASC and CBBDQ total and by dimension scores. Cronbach’s alpha was used to test the reliability of the scales. A p-value <0.05 was considered statistically significant.

### Ethical considerations

Before starting the study, we obtained permission by e-mail to use the scales. The study was approved by the Health Ethics Committee of Sakarya University (04/04/2020-E.3950) and the Provincial Directorate of National Education (16/10/2020). Verbal permission was also obtained from the administrators of the schools where the study was conducted. We informed the children and their mothers about the research and assured them of the protection of their personal information. Verbal and written consent was obtained in accordance with the volunteerism principle.

## Results

We found that 72.5% of the children were nine years old and 27.5% were ten, 49.6% were girls, and 50.4% were boys. Among the mothers of the children, 39.7% were high school graduates, and 73% stated their income and expense levels were equivalent. Four-point-seven percent of the children were underweight, 57.9% were of normal weight, 19.6% were overweight, and 17.9% were obese. The rate of incontinence experienced by the children while watching videos was 3.6%.

Some sociodemographic characteristics of the included children and their mothers are presented in table 1. The examination results of the relationship between some sociodemographic characteristics and the VASC, the CBBDQ, and the dimension scores of the participants are shown in table 1. No significant difference was found between the children’s total VASC and CBBDQ and dimension scores based on their age (p > 0.05). The total VASC scores of the boys and their self-control, reward-reinforcement, problems, and involvement dimension scores were significantly higher than the scores of the girls (p < 0.05). Comparing maternal education levels, the VASC total, rewardreinforcement dimension, and involvement dimension scores of the children with high school graduate mothers were significantly lower than to those of children with primary-middle school or university graduate mothers. The VASC total and self-control dimension scores of the children whose families had lower income levels were higher (table 1). The median bowel subscale score of the children with primary-middle education mothers was significantly higher than that of children with high school education mothers. Bladder and bowel symptoms were significantly higher in children whose mothers had lower income than expense levels (p < 0.05).

**Table 1:** Comparison between the VASC scores and some sociodemographic characteristics of the children and their mothers (N = 363)

Sociodemographic characteristics		n (%)	Self-control	Reward-reinforcement	Problems	Involvement	Total score
Age (years)							
	9	263 (72.5)	17.0 (7.0 - 33.0)	20.0 (6.0 - 28.0)	7.0 (4.0 - 20.0)	7.0 (4.0 - 20.0)	50.52 ± 15.82
	10	100 (27.5)	17.0 (7.0 - 35.0)	20.0 (6.0 - 27.0)	7.0 (4.0 - 18.0)	7.5 (4.0 - 19.0)	51.44 ± 17.13
Test statistics			Z = -0.029	Z = -0.033	Z = -1.404	Z = -0.646	t = -0.483
			p = 0.977	p = 0.974	p = 0.160	p = 0.518	p = 0.629
Child’s gender							
	Female	180 (49.6)	14.0 (7.0 - 33.0)	18.0 (6.0 - 27.0)	6.0 (4.0 - 19.0)	7.0 (4.0 - 13.0)	46.85 ± 15.15
	Male	183 (50.4)	19.0 (7.0 - 35.0)	21.0 (6.0 - 28.0)	8.0 (4.0 - 20.0)	8.0 (4.0 - 20.0)	54.63 ± 16.26
Test statistics			Z = -4.389	Z = 2.954	Z = -4.428	Z = -3.439	t = -4.716
			p = 0.000***	p = 0.003**	p = 0.000***	p = 0.001**	p = 0.000***
Mother’s education							
	Primary-middle school (1)	106 (29.2)	17.0 (7.0 - 35.0)	20.0 (6.0 - 28.0)	7.5 (4.0 - 19.0)	8.0 (4.0 - 14.0)	52.42 ± 15.46
	High school (2)	144 (39.7)	15.0 (7.0 - 33.0)	17.0 (6.0 - 27.0)	7.0 (4.0 - 19.0)	7.0 (4.0 - 18.0)	47.21 ± 14.53
	University (3)	113 (31.1)	17.0 (7.0 - 34.0)	21.0 (6.0 - 27.0)	7.0 (4.0 - 20.0)	8.0 (4.0 - 20.0)	53.76 ± 18.00
Test statistics			KW = 4.362	KW = 18.775	KW = 4.541	KW = 7.607	F = 6.130
			p = 0.113	p = 0.000***	p = 0.103	p = 0.022*	p = 0.002**
				1 > 2^a^; 3 > 2^a^		3 > 2 a	1 > 2^b^; 3 >2^b^
Economic status							
	Income is less than expenses (1)	57 (15.7)	19.0 (7.0 - 35.0)	20.0 (6.0 - 28.0)	8.0 (4.0 - 19.0)	7.0 (4.0 - 19.0)	53.44 ± 18.14
	Income is equivalent to expenses (2)	265 (73)	17.0 (7.0 - 33.0)	20.0 (6.0 - 27.0)	7.0 (4.0 - 20.0)	8.0 (4.0 - 20.0)	51.26 ± 16.13
	Income is more than expenses (3)	41 (11.3)	13.0 (7.0 - 28.0)	17.0 (6.0 - 26.0)	6.0 (4.0 - 15.0)	6.0 (4.0 - 11.0)	43.87 ± 11.42
Test statistics			KW = 11.733	KW = 3.461	KW = 4.139	KW = 4.216	F = 4.716
			p = 0.003**	p = 0.177	p = 0.126	p = 0.122	p = 0.010*
			1 > 2 ^a^; 2 > 3^a^				1 > 2 ^b^; 2 > 3^b^
Weight status category							
	Underweight (1)	17 (4.7)	19.0 (7.0 - 33.0)	20.0 (6.0 - 26.0)	9.0 (4.0 - 19.0)	8.0 (4.0 - 13.0)	56.52 ± 20.28
	Normal or healthy weight (2)	210 (57.9)	17.0 (7.0 - 33.0)	19.0 (6.0 - 27.0)	7.0 (4.0 - 20.0)	7.0 (4.0 - 20.0)	50,64 ± 16,47
	Overweight (3)	71 (19.6)	17.0 (7.0 - 33.0)	20.0 (8.0 - 28.0)	7.0 (4.0 - 17.0)	8.0 (4.0 - 14.0)	52.22 ± 14.63
	Obese (4)	65 (17.9)	15.0 (7.0 - 35.0)	19.0 (6.0 - 27.0)	7.0 (4.0 - 18.0)	7.0 (4.0 - 19.0)	48.09 ± 15.42
Test statistics			KW = 1.976	KW = 3.022	KW = 7.489	KW = 4.585	F = 1.514
			p = 0.577	p = 0.388	p = 0.058	p = 0.205	p = 0.211
Eating or snacking while watching a video							
	Yes	181 (49.9)	19.0 (7.0 - 34.0)	21.0 (6.0 - 28.0)	8.0 (4.0 - 20.0)	8.0 (4.0 - 20.0)	54.59 ± 16.55
	No	182 (50.1)	14.0 (7.0 - 35.0)	18.0 (6.0 - 27.0)	6.0 (4.0 - 18.0)	7.0 (4.0 - 17.0)	46.97 ± 14.89
Test statistics			Z = -4.187	Z = -3.498	Z = -3.552	Z = -3.061	t = -4.613
			p = 0.000***	p = 0.000***	p = 0.000**	p = 0.002**	p = 0.000***
Incontinence while watching videos							
	Yes	13 (3.6)	19.0 (13.0 - 33.0)	22.0 (11.0 - 27.0)	10.0 (4.0 - 13.0)	8.0 (5.0 - 11.0)	58.84 ± 12.16
	No	350 (94.6)	17.0 (7.0 - 35.0)	20.0 (6.0 - 28.0)	7.0 (4.0 - 20.0)	7.0 (4.0 - 20.0)	50.47 ± 16.24
Test statistics			Z = -2.077	Z = -1.721	Z = -2.333	Z = -0.233	t = 1.838
			p = 0.038*	p = 0.085	p = 0.020*	p = 0.816	p = 0.067

VASC: Video Game Addiction Scale for Children; KW: Kruskal-Wallis H Test; Z: Mann-Whitney U-Test; F: One way ANOVA; t: Independent Sample t-test; ^a^ Tamhane’s T2 test; ^b^ Tukey ANOVA test

*p < 0.05; **p < 0.01; ***p < 0.001

As shown in tables 1 and 2, a statistically significant relationship was found between urinary/fecal incontinence while watching videos and the total VASC, self-control dimension, problems dimension, total CBBDQ, and bladder subscale scores of the participants (p < 0.001). The children who had a meal or snack while watching videos had significantly higher total VASC, self-control, reward-reinforcement, problems, and involvement scores (p < 0.05) (table 1). Overweight children had more bladder symptoms compared to normal-weight children (table 2).

**Table 2:** The comparison between the CBBDQ scores and some sociodemographic characteristics of the children and their mothers (N = 363)

Sociodemographic characteristics		n (%)	CBBDQ		
			Bladder Dysfunction	Bowel Dysfunction	Total score
Age (years)					
	9	263 (72.5)	16.0 (10.0 - 45.0)	12.0 (8.0 - 32.0)	29.0 (19.0 - 70.0)
	10	100 (27.5)	16.0 (10.0 - 47.0)	12.0 (8.0 - 36.0)	29.0 (18.0 - 73.0)
Test statistics			Z = -0.721	Z = -0.151	Z = -0.568
			p = 0.471	p = 0.880	p = 0.570
Child’s gender					
	Female	180 (49.6)	15.0 (10.0 - 45.0)	12.0 (8.0 - 36.0)	28.0 (18.0 - 70.0)
	Male	183 (50.4)	17.0 (10.0 - 47.0)	12.0 (8.0 - 34.0)	29.0 (18.0 - 73.0)
Test statistics			Z = -1.728	Z = -1.279	Z = -1.798
			p = 0.084	p = 0.201	p = 0.072
Mother’s education					
	Primary-middle school (1)	106 (29.2)	17.0 (10.0 - 45.0)	13.0 (8.0 - 36.0)	29.0 (18.0 - 70.0)
	High school (2)	144 (39.7)	16.0 (10.0 - 47.0)	12.0 (8.0 - 26.0)	29.0 (18.0 - 73.0)
	University (3)	113 (31.1)	16.0 (10.0 - 41.0)	13.0 (8.0 - 27.0) p = 0.044*	28.0 (18.0 - 58.0)
Test statistics			KW = 2.510	KW = 6.228	KW = 1.953
			p = 0.285	p = 0.044*	p = 0.377
				1 > 2^a^	
Economic status					
	Income is less than expenses (1)	57 (15.7)	19.0 (10.0 - 45.0)	14.0 (8.0 - 34.0)	32.0 (18.0 - 70.0)
	Income is equivalent to expenses (2)	265 (73)	16.0 (10.0 - 47.0)	12.0 (8.0 - 36.0)	28.0 (18.0 - 73.0)
	Income is more than expenses (3)	41 (11.3)	14.0 (10.0 - 27.0)	12.0 (8.0 - 18.0)	28.0 (18.0 - 38.0)
Test statistics			KW = 10.310	KW = 5.911	KW = 10.813
			p = 0.001**	p = 0.015*	p = 0.001**
			1 > 2^a^; 1 > 3^a^	1 > 3^a^	1 > 2^a^ ; 1 > 3^a^
Weight status category					
	Underweight (1)	17 (4.7)	18.0 (10.0 - 32.0)	12.0 (8.0 - 16.0)	31.0 (18.0 - 46.0)
	Normal or healthy weight (2)	210 (57.9)	15.0 (10.0 - 45.0)	12.0 (8.0 - 32.0)	28.0 (18.0 - 70.0)
	Overweight (3)	71 (19.6)	17.0 (10.0 - 30.0)	14.0 (8.0 - 36.0)	31.0 (18.0 - 66.0)
	Obese (4)	65 (17.9)	17.0 (10.0 - 47.0)	12.0 (8.0 - 34.0)	29.0 (18.0 - 73.0)
Test statistics			KW = 3.315	KW = 7.923	KW = 6.508
			p = 0.346	p = 0.048*	p = 0.089
				3 > 2^b^	
Eating or snacking while watching a video					
	Yes	181 (49.9)	17.0 (10.0 - 45.0)	12.0 (8.0 - 36.0)	30.0 (18.0 - 70.0)
	No	182 (50.1)	15.0 (10.0 - 47.0)	12.0 (8.0 - 34.0)	28.0 (18.0 - 73.0)
Test statistics			Z = 1.185	Z = -0.126	Z = -1.081
			p = 0.236	p = 0.899	p = 0.280
Incontinence while watching videos					
	Yes	13 (3.6)	25.0 (16.0 - 47.0)	17.0 (8.0 - 27.0)	42.0 (27.0 - 73.0)
	No	350 (94.6)	16.0 (10.0 - 41.0)	12.0 (8.0 - 36.0)	29.0 (18.0 - 76.0)
Test statistics			Z = -4.435	Z = -1.811	Z = -4.012
			p = 0.000***	p = 0.070	p = 0.000***

CBBDQ: Childhood Bladder and Bowel Dysfunction Questionnaire; KW: Kruskal-Wallis H test; Z: Mann-Whitney U test

^a^ Tamhane’s T2 test

^b^ Games-Howel Test

* p < 0.05

** p < 0.01

*** p < 0.001

 The total VASC and CBBDQ, and dimension mean scores of the participants are shown in table 3. The VASC mean score of the children was 50.77 ± 16.17, whereas the CBBDQ mean score of the mothers was 29.98 ± 8.90. The ratio of children with a mean score equal to or greater than 90 on VASC was 0.8% (n = 3).

**Table 3:** The correlation between VASC and CBBDQ scores (n = 363)

Scales	CBBDQ							
		Descriptive	CBBDQ total score		Bladder dysfunction		Bowel dysfunction	
	Range	Mean ± SD	r	p	r	p	r	p
VASC	21 - 98	50.77 ± 16.17	0.220	0.000***	0.202	0.000***	0.168	0.001**
Total score	7 - 35	16.88 ± 7.29	0.192	0.000***	0.181	0.001**	0.141	0.007**
Self-control	6 - 28	18.45 ± 5.56	0.171	0.001**	0.154	0.003**	0.135	0.010**
Reward-reinforcement Problems	4 - 20	7.58 ± 3.49	0.231	0.000***	0.222	0.000***	0.162	0.002**
Involvement	4 - 20	7.85 ± 2.85	0.139	0.008**	0.113	0.031*	0.129	0.014*

VASC: Video Game Addiction Scale for Children; CBBDQ: Childhood Bladder and Bowel Dysfunction r: Correlation coefficient

* p < 0.05

** p < 0.01

*** p < 0.001

A weak positive correlation was found between the VASC scores of the participants and their CBBDQ (r = 0.220; p = 0.000), bladder subscale (r = 0.202; p = 0.000), and bowel subscale (r = 0.168; p = 0.001) scores (table 3).

## Discussion

In this section, the relationship between children’s video game viewing habits and bladder/bowel dysfunctions is discussed based on the literature. The associations between some characteristics of the participants and their VASC total and dimension scores are shown in table 1. The mean total VASC score of the boys was 54.63 ± 16.26, the mean total VASC score of the girls was 46.85 ± 15.15, and the difference between them was significant.

This result was one of the remarkable findings of the study. In the study conducted by De Pasquale *et al*. (2021), the mean total VASC score was 51.63 for boys and 42.20 for girls ^17^. Many studies examining video game addiction have supported our result, and a relationship between the male gender and higher VASC scores has been reported ^18^^-^^20^. In contrast, another study found that girls had higher scores and were more at risk of addiction ^21^. In our sample, three male children (0.8%) scored 90 or above and should be examined in detail in terms of addiction. In the study carried out by Oflu and Yalçin (2019) using VASC, they reported four children with a score of 90 or above (1.6%), all of them were boys ^22^. Apparently, boys are more prone to video game addiction, probably explained by different physical, psychological, and personality traits between boys and girls.

There were significant relationships between the VASC scores of the children and the education levels of their mothers, the income levels of their families, their body mass index (BMI) category, their statuses of eating or snacking while watching videos, and their experiences of urinary/fecal incontinence while watching videos (table 1). Previous studies showed that as the education and income levels of the mother’s decrease, their children’s time using the internet and playing video games increases ^23^^,^^24^. This result may be associated with the mothers’ lack of knowledge about the dangers of prolonged use of technology. Similar to this result, in the literature, snacking on sweet and high-calorie foods during the time spent on sedentary activities such as watching TV, computer, and internet use increased the risk of obesity ^25^^,^^26^.

A significant relationship was determined between the CBBDQ scores of the participants and their experiences of urinary/fecal incontinence while watching videos (table 2). Significant associations have been found in previous studies between playing video games for prolonged periods and high levels of depression, aggression, trait anxiety, emotional instability, impaired family communication, loss of appetite, sleep disorders, and behaviors of neglecting or being reluctant to take part in physical activities ^27^^-^^29^.

In addition to the problems observed in previous studies, child’s prolonged presence in front of a screen causes a decrease in their physical activity levels and delays in their urination/defecation. In our study, the ratio of children experiencing urinary incontinence while watching videos was 3.6%, which is worth noting. In a prevalence study, day-night incontinence and constipation were the most common symptoms of bladder-bowel dysfunction, and the postponement of urination/defecation by children who intentionally squeeze their sphincters despite needing to urinate contributed to the development of bladder-bowel dysfunction ^15^^,^^30^. Children at risk should be referred to the relevant units and analyzed physically and psychologically.

The relationship between the VASC and CBBDQ scores of the participants was analyzed with a correlation analysis. We found significant positive relationships between the VASC self-control and total CBBDQ and bladder subscale scores; and between total VASC and problems dimension scores (table 3). As the time of screen use increases, children sleep less, their blood pressure increases, their HDL cholesterol levels decrease, and insulin resistance increases, causing visual disorders, obesity, and many other health problems associated with inactivity ^31^. Furthermore, there is also a decrease in children’s school success due to decreased attention and memory capacity ^32^.

In this study, we examined the relationships between excretion status and video game addiction and observed that video game addiction adversely affected excretion. Parents should impose time limitations on children’s technology use. We think being in front of a screen for a long time affects the excretory system, and the child delays going to the toilet. Physical activity should be encouraged in children to discharge their energy.

The results of this study can be generalized to children studying at the schools where we collected the data. It is difficult to measure video game addiction. The measurements in this study were performed based on the children self-reports. The children’s BMI values were calculated according to the height and weight information provided by them and their parents. The inability of the researchers to measure children’s height and weight was among the limitations of the study. Since primary and secondary schools are separated, we included only children in the 9-10 years age group from primary schools. The study’s strength is being the first study to examine the relationship between bladder and bowel dysfunction and video game addiction in children.

In this study, the ratio of children who experienced urinary/fecal incontinence while playing video games was significant, and it was a remarkable result that all three children at risk of video game addiction were male. Increased video game addiction scores were associated with increased bladder and bowel dysfunction scores.

These results will help understanding the impact of video game addiction on bladder and bowel dysfunction by drawing a framework. Furthermore, the resulting evidence showed variables affecting both video game addiction and bladder/bowel dysfunction. We think that interventions should be made to reduce video game addiction, which was the independent variable in this study. Accordingly, it is important to provide awareness training for children and parents on video game playing duration, healthy internet use, and the harms of internet addiction.

A detailed evaluation of children’s video game habits and potential bladder-bowel dysfunction patterns, identifying at-risk children, and directing them to the relevant units are extremely important for the prevention of health conditions that may adversely affect their lives. Moreover, schools should contribute to the children’s physical and mental health development by organizing after-school activities that support their development (e.g., physical activities or active games). We recommend conducting studies to evaluate different intervention effects in children at risk, such as education and counseling programs.
